# Lessons from the CONSCIOUS-1 Study

**DOI:** 10.3390/jcm9092970

**Published:** 2020-09-14

**Authors:** Alexander J. Schupper, Matthew E. Eagles, Sean N. Neifert, J Mocco, R. Loch Macdonald

**Affiliations:** 1 Department of Neurosurgery, Icahn School of Medicine at Mount Sinai, New York, NY 10029, USA; alexander.schupper@mountsinai.org (A.J.S.); sean.neifert@icahn.mssm.edu (S.N.N.); j.mocco@mountsinai.org (J.M); 2 Department of Clinical Neurosciences, Division of Neurosurgery, Alberta Children’s Hospital, University of Calgary, Alberta, AB T3B 6A8, Canada; meagles@mun.ca; 3 Department of Neurological Surgery, UCSF Fresno, Fresno, CA 93701, USA

**Keywords:** angiographic vasospasm, cerebral aneurysm, cerebral infarction, delayed cerebral ischemia, management, subarachnoid hemorrhage

## Abstract

After years of research on treatment of aneurysmal subarachnoid hemorrhage (aSAH), including randomized clinical trials, few treatments have been shown to be efficacious. Nevertheless, reductions in morbidity and mortality have occurred over the last decades. Reasons for the improved outcomes remain unclear. One randomized clinical trial that has been examined in detail with these questions in mind is Clazosentan to Overcome Neurological Ischemia and Infarction Occurring After Subarachnoid Hemorrhage (CONSCIOUS-1). This was a phase-2 trial testing the effect of clazosentan on angiographic vasospasm (aVSP) in patients with aSAH. Clazosentan decreased moderate to severe aVSP. There was no statistically significant effect on the extended Glasgow outcome score (GOS), although the study was not powered for this endpoint. Data from the approximately 400 patients in the study were detailed, rigorously collected and documented and were generously made available to one investigator. Post-hoc analyses were conducted which have expanded our knowledge of the management of aSAH. We review those analyses here.

## 1. Introduction

Subarachnoid hemorrhage (SAH) is a rare, albeit dangerous form of stroke, most commonly occurring due to a ruptured aneurysm (aSAH) [1]. Evidence from randomized, clinical trials (RCT) that guides treatment of aSAH is oral nimodipine and securing the ruptured aneurysm, with endovascular coiling recommended over surgical clipping in aneurysms that are amenable to both methods [2,3,4,5]. These patients can have other complications since they are often critically ill and subjected to intensive care and other medical and surgical management for days after the hemorrhage [6]. Angiographic vasospasm (aVSP) and delayed cerebral ischemia (DCI) are among the most serious complications and are unique to this disease [7,8]. Many treatments for aSAH have been investigated, including about 30 in double-blind RCT [9].

One RCT was the Clazosentan to Overcome Neurological iSChemia and Infarction OccUrring after Subarachnoid hemorrhage (CONSCIOUS-1). This phase-2 study found that clazosentan reduced moderate or severe aVSP [10]. This study was not designed to detect differences between groups in a three-month clinical outcome. The subsequent CONSCIOUS-2 and CONSCIOUS-3 trials were unsuccessful. Actelion Pharmaceuticals generously made the CONSCIOUS-1 data available to one of the investigators [11,12]. Secondary, post-hoc analyses of the data have yielded new information about treatment of patients with aSAH. Herein we describe the knowledge gained from these secondary analyses.

## 2. CONSCIOUS-1

CONSCIOUS-1 was a randomized, double-blinded, placebo-controlled phase-2 dose-finding study to determine the safety and efficacy of clazosentan, an endothelin receptor antagonist, in preventing aVSP after aSAH [10]. The trial randomized 413 patients to receive placebo or one of 3 doses of clazosentan beginning within 56 h of aSAH. The primary endpoint was moderate or severe aVSP, assessed by centralized, blinded review of digital subtraction angiograms (DSA) done at baseline prior to aneurysm repair and 7–11 days post-aSAH. All patients underwent cranial computed tomography (CT) within 48 h of SAH, 24–48 h after the aneurysm repair and then at 6 weeks. The secondary endpoint was a composite of death from any cause within 6 weeks, new cerebral infarct within 6 weeks compared to the post-procedural CT scan, delayed ischemic neurological deficit (DIND, defined basically the same as DCI) due to VSP within 14 days and rescue therapy for DSA or transcranial Doppler VSP within 14 days [13]. Clinical outcome was assessed with the extended GOS [14]. The study found a dose-dependent reduction in moderate or severe aVSP. There was no significant effect on the composite secondary outcome. Clazosentan was associated with pulmonary complications, hypotension and anemia, all which were deemed to be minor adverse effects. The effect of aVSP on other patient outcomes and inpatient healthcare resource use was assessed [12]. The reduction in severe aSVP was associated with improvement in the mini-mental state, EuroQol Group-5D and functional status exam as well as reduced hospital and intensive care unit length of stay.

CONSCIOUS-1 spawned a two-phase 3 RCT testing of clazosentan in aSAH patients undergoing clipping or coiling [8,11,12]. Minimal changes were made to the protocols since clazosentan had achieved its pharmacologic effect to reduce aVSP in CONSCIOUS-1. The main difference was the primary endpoint which was VSP-related morbidity and all-cause mortality within 6 weeks of aSAH, defined as: death; aVSP-related cerebral infarction (where aVSP was the primary cause or a relevant contributing factor); DIND due to aVSP (where aVSP was the primary cause or a relevant contributing factor); or neurological signs or symptoms, in the presence of DSA showing aVSP, leading to rescue therapy. The CONSCIOUS-2 and -3 RCT did not show an effect of clazosentan on this endpoint [11,12].

## 3. Aneurysm Repair in CONSCIOUS-1

The International Subarachnoid Aneurysm Trial (ISAT) found outcome after aSAH was better with endovascular coiling for aneurysms deemed amenable to endovascular or open surgical repair [4]. Understanding why coiling led to better outcomes could generate hypotheses about ways to improve the results of clipping, as well as outcome after aSAH in general. We analyzed aVSP and DIND in C1 patients who received clipping or coiling [15]. The CONSCIOUS-1 data are especially useful for analyzing this issue because every patient had DSA and CT scanning so there is no bias in selection of patients for imaging. Propensity score matching was used in most of the studies described here in order to account as best as possible for the fact that patients were not randomized to aneurysm repair method or to anything other than clazosentan or placebo [15,16]. Endovascular coiling was associated with less aVSP and DIND compared to surgical clipping, but there was no difference between the repair methods in delayed cerebral infarction or clinical outcome. De Oliveira et al. reviewed the literature up until 2007 and found no difference in VSP between clipped and coiled patients, although the definition of VSP varied and most studies were single center, retrospective reviews [17]. Less aVSP and DIND after coiling is consistent with data from the only RCT of clipping versus coiling [18]. This difference would also contribute to the benefit of coiling over clipping.

About 50% of patients in CONSCIOUS-1 had hypodensities on CT scan and many appeared between the admission and immediate post-aneurysm repair CT scans [16]. In addition to aVSP, DIND and delayed cerebral infarcts, we compared early versus late cerebral infarcts in patients undergoing surgical clipping and endovascular coiling [16]. Clipping was found to be an independent risk factor for early cerebral infarction. As expected, delayed infarcts were associated with aVSP. Early cerebral infarcts were found to be stronger predictors of poor outcome than late infarcts, which would also be to the detriment of clipping as compared to coiling.

The most important factors leading to aVSP are the location, volume, concentration (density on CT scan) and duration of presence of SAH [19]. Since there was less aVSP and DIND in coiled patients in CONSCIOUS-1, we compared clot clearance (in terms of Hijdra score) between patients undergoing surgical clipping or endovascular coiling as a possible reason for the differences in outcomes between the two repair methodologies [20,21]. There was no difference in clot clearance between groups, despite coiling being associated with less severe aVSP. That the clot clearance measurements were meaningful was supported by the finding that faster clot clearance in general was associated with less severe aVSP. We concluded clot clearance was unlikely to be a driver of differences in outcomes between coiling and clipping.

Another explanation for the difference in outcomes between clipping and coiling could be aneurysm repair complications [22]. Perioperative complications were compared between endovascular coiling and surgical clipping in CONSCIOUS-1. Prospective and independently verified adverse events and imaging and use of propensity score matching in this study are strengths compared to most of the other studies comparing clipping and coiling, which are retrospective or even prospective self-assessed and usually single center studies. Patients who received clipping had a larger decline in their Glasgow coma score after surgery compared to patients undergoing coiling and this decline was associated with worse clinical outcome. Perioperative thromboembolism was associated with postoperative Glasgow coma score decrease in patients receiving coiling whereas in clipped patients the decreased consciousness was associated with intraoperative hypertension or hypotension. Retrospective studies published mainly subsequently suggest that antiplatelet drugs reduce DCI after coiling, which is consistent with a detrimental role of thromboembolic events after coiling [23]. A Cochrane review found 7 RCT of antiplatelet drugs in patients with aSAH [24]. No significant effects of antiplatelet drugs were found, although there were trends towards reduced DCI, poor outcome and increased intracranial hemorrhage. Regarding blood pressure management, hypotension is widely thought to be detrimental. Even lowering blood pressure to prevent rebleeding prior to aneurysm repair has not been as safe and effective as expected and induced hypertension also has little evidence to support its use [25,26,27,28]. The dangers of high or low blood pressure during aneurysm clipping need to be remembered based on this data.

## 4. Critical Care Management of aSAH

Predictors of prolonged intensive care stay following aSAH were older age, history of hypertension, and World Federation of Neurosurgical Societies (WFNS) Score 4 or 5 [29]. These findings are unsurprising and reflect that they are also the three most important prognostic factors for overall outcome [30]. Complications or factors that are associated with intensive care stay have not been well studied in the CONSCIOUS-1 data. The most frequent medical complications of aSAH in one large prospective observational study were temperature > 38.3 °C (54%), anemia treated with transfusion (36%) and hyperglycemia > 11.1 mmol/L (30%) [31]. Fever, anemia and transfusion have been investigated in CONSCIOUS-1 [32,33,34]. Fever often cannot be attributed to infection and may be centrally mediated in the brain or due to noninfectious inflammation. A manifestation of the latter could be the systemic inflammatory response syndrome (SIRS). We identified 3 studies that found 29% to 87% of patients with SAH develop SIRS (overall 58% of 621 patients) [35,36,37]. In CONSCIOUS-1, 63% of patients developed SIRS [38]. SIRS was associated with poor outcome which was consistent with the prior publications. However, we found no association of SIRS with aVSP, DIND or cerebral infarction, in contrast to prior studies.

Septic and critically ill patients in general can develop cognitive impairment in the absence of cerebral infarcts [39]. A quarter of 821 patients admitted to intensive care with respiratory failure or shock had global cognitive scores one year later that were similar to patients with mild Alzheimer disease, compared to 6% before they were admitted [40]. It has been hypothesized that some of the cognitive impairment in survivors of aSAH could be due to similar pathophysiology to that occurring is other critically-ill patients and that this could be detected in generalized brain volume loss in the absence of focal cerebral infarctions or hypodensities [41,42]. We measured brain volumes of 97 patients in CONSCIOUS-1 who had no focal brain hypodensities on CT scan. Predictors of and the effect of post-SAH cerebral atrophy on outcomes were assessed. Older age, male sex and SIRS were associated with global cerebral atrophy [32]. Brain atrophy was associated with worse modified Rankin (mRS), National Institutes of Health Stroke Scale and EuroQol Group-5D scores as well as lower executive functioning [43,44].

Inflammation is hypothesized to contribute to DCI and poor outcome after aSAH and the data on SIRS, while showing an association and not causation, support this theory [45,46,47]. Effects of anti-inflammatory drugs including glucocorticoid steroids on DCI and outcome after aSAH are reported in uncontrolled studies from which no conclusions can be drawn [48,49]. Patients recorded as taking acetylsalicylic acid prior to admission for aSAH and undergoing endovascular aneurysm repair in the National Inpatient Sample had shorter hospital stay and less likelihood of nonroutine discharge [50]. There are at least 3 RCT of acetylsalicylic acid in patients with SAH [24]. They were included in a Cochrane review of 7 RCT of antiplatelet drugs in patients with aSAH [24]. No significant effects of antiplatelet drugs were found, although there were trends towards reduced DCI and poor outcome and increased intracranial hemorrhage. Other uncontrolled studies suggest antiplatelet drugs reduce DCI and may improve outcome after aSAH but mainly in coiled patients [51]. A propensity-score matched study of the use of nonsteroidal anti-inflammatory agents was conducted on patients in CONSCIOUS-1 [33]. These drugs were associated with reduced intensive care stay and a trend towards improved outcome (81% versus 69% good outcome) but no salient effects on aVSP or DIND. One RCT evaluated a nonsteroidal anti-inflammatory drug meloxicam in SAH and showed no differences in in-hospital mortality or GOS on discharge, with a slight trend toward reduced middle cerebral artery transcranial Doppler flow velocity in patients treated with meloxicam [52].

Anemia and transfusion are common after aSAH and were reviewed by Leroux [53]. Hemodilution used to be recommended to improve cerebral blood flow but now it is believed that it increases cerebral blood flow simply to maintain rather than improve brain oxygen delivery [54]. Indeed, anemia has been associated with poor outcome, although transfusion has as well [55]. Reasons to avoid anemia are that it could reduce oxygen delivery to the brain. On the other hand, increased hemoglobin concentration theoretically could impair cerebral blood flow by increasing blood viscosity. Avoiding anemia could avoid the need for transfusion, which has deleterious effects such as organ injury and increased risk of infection [55]. Transfusion, however, been shown by positron emission tomography to be one of the best methods to increase brain oxygen delivery [54]. We analyzed anemia, defined as hemoglobin < 10 g/dL, at different times after aSAH (at hospital admission before aneurysm securing, one to 3 days post-aneurysm securing and during the peak DCI window 5 to 9 days after admission) in CONSCIOUS-1 [34]. Anemia after aneurysm securing and during the DCI window was associated with poor neurologic outcome, while anemia after aneurysm securing was associated with mortality. In a propensity score matched analysis, transfusion of anemic patients had no effect on outcome whereas transfusing patients with hemoglobin > 10 g/dL was associated with improved neurological outcome. Even with propensity score matching, biases cannot be excluded. A RCT of transfusion in aSAH is being conducted (ClinicalTrials.gov Identifier: NCT03309579). It is best to avoid anemia; minimizing iatrogenic blood loss is highly recommended.

In addition to maintaining some as yet unknown optimal hemoglobin concentration, intravascular volume and blood pressure are intensely focused on in patients with aSAH. Guidelines recommend maintaining euvolemia [56,57,58]. Measuring and achieving this is notoriously inexact and fluid management after aSAH varies between centers [59,60]. The effect of fluid balance and colloid use on outcomes following SAH was analyzed in the CONSCIOUS-1 data [61]. DIND was associated with higher fluid input but not with fluid balance. The detrimental effect of hypervolemia was probably detected in this study as higher fluid input since measuring fluid input is easier and more accurate whereas measuring fluid balance is harder to do accurately [62]. Ibrahim and colleagues also reported colloid administration and positive fluid balance were associated with poor outcome [61]. The findings are consistent with conclusions of van der Jagt’s review, three years later, of literature on fluid management of critically-ill brain injured patients [63]. He concluded that the best hypothesis was that these patients should be kept euvolemic through administration of non-colloid fluids in an attempt to maintain normal physiologic conditions.

One hypothesis that emerges from the above results is that the best outcome for patients with aSAH may be achieved by keeping all of the patient’s clinical chemistry, vital signs and other physiologic parameters normal because if they deviate from this then an intervention is done to correct it and that intervention has risks which potentially add to the harmful effects of the initial problem. Also, there is a glaring lack of RCT evidence investigating the associations of these medical complications and their treatment, so the causality and relative risks and benefits involved are largely unknown. 

The effect on fluctuations in sodium in patients with aSAH in CONSCIOUS-1 are consistent with this concept of the negative effects of nonphysiologic conditions after aSAH [64]. Changes in daily sodium were associated with poor outcome, and the absolute daily difference in sodium during the DCI window trended toward significance as an independent predictor of DCI. Hyponatremia at some time after aSAH in 33% (33/101) of patients in one series [65]. Intravenous high sodium fluids are increasingly administered to these patients and sometimes mineralocorticoids are also prescribed, despite lack of benefit of the latter in RCT [66]. These interventions make it difficult to disentangle the effects of serum sodium changes from the treatments for it. While hypernatremia in CONSCIOUS-1 has not been examined, targeting higher serum sodium concentrations after brain injury has been increasingly applied for intracranial pressure management. It is not at all clear, however, what the effects of this are on functional outcome [67].

Other aSAH management controversies include seizure prophylaxis and external ventricular drain management. The predictors and effects of seizures following SAH on functional outcomes were analyzed in CONSCIOUS-1 [68]. A poor SAH grade and midline shift or subdural hematoma on admission CT scan were associated with seizures in univariate analysis. On multivariable analysis, subarachnoid clot burden and subdural hematoma were associated with post-SAH seizures. Predicting seizures is important particularly if one wants to tailor prophylaxis to patients who are at higher risk of seizures. What patients should receive seizure prophylaxis is the key question, which has never been studied in RCT. We also examined shunt-dependent hydrocephalus in CONSCIOUS-1 [69]. Seventeen percent of patients required shunt placement. This frequency varies greatly among institutions; it is not clear what the basis for these variations is. In CONSCIOUS-1, use of an external ventricular drain and increased daily cerebrospinal fluid output predicted shunt placement.

Acute kidney injury has seldom been studied in aSAH. Eagles et al. analyzed acute kidney injury and outcome in CONSCIOUS-1 [64]. History of hypertension and nephrotoxic medications were independent predictors of developing acute kidney injury. Acute kidney injury was an independent predictor of death, but not a functional outcome.

## 5. Angiographic VSP, DCI and Outcome

Intense resources are directed at assessing patients for their risk of DCI. Efforts have been made to tailor postoperative vigilance and monitoring and guide prophylactic treatments to patients who are at highest risk. Since the best predictor of DCI is volume of SAH, several investigators measured SAH volume on CT. Quantitative clot volumes generally predict DCI better than scales like the modified Fisher scale but are still of only moderate predictive value [70,71]. This is probably because the concentration of blood and how long it stays in the subarachnoid space are also important [19]. Intraventricular hemorrhage also spills blood into the subarachnoid space and contributes to DCI, hence its inclusion in the modified Fisher scale. The definition of intraventricular hemorrhage in the modified Fisher scale was unclear. We studied whether a semiquantitative measurement of intraventricular hemorrhage, the modified Graeb score, would improve the prediction of DCI by the modified Fisher scale [72]. Both of these scales had similar predictability for DCI and the combination of the two was slightly better [73]. 

Another factor influencing prediction of DCI is interobserver variability of assessment of these variables. We found in CONSCIOUS-1 that the interobserver agreement for the Fisher and modified Fisher scales on the initial CT scans was fair to moderate (*kappa* 0.41; 95% confidence interval (CI) 0.33–0.49) [71,74]. There was better agreement when semiquantitative scales like Graeb and Hijdra scales were used (intraclass correlation coefficient (ICC) 0.56 (95% CI 0.49–0.62)) [21,72]. Similarly, there was poor agreement in diagnosing hydrocephalus but excellent agreement when measuring the ventriculocranial ratio (ICC 0.77; 95% CI 0.72–0.81). These findings are consistent with prior studies comparing qualitative and quantitative methods of CT assessment [75,76].

Adding clinical and laboratory parameters may improve DCI prediction although some of these factors like age, aneurysm location, cigarette smoking and preexisting hypertension are inconsistently found to relate to DCI [77,78].

De Oliveria Manoel and colleagues created a grading scale, entitled VASOGRADE, and assessed it for its ability to predict DCI following SAH [79]. Data from CONSCIOUS-1 and two other phase 2 studies was used [80,81]. The VASOGRADE categorized patients into three groups using admission neurological grade and modified Fisher scale. The VASOGRADE had adequate discrimination for prediction of DCI (area under the receiver operating characteristics curve = 0.63) and good calibration.

One consequence of CONSCIOUS-1 seems to have been resurgence of opinion that aVSP is not the sole or even the most important cause of DCI [9,82,83]. There also has more emphasis on early brain injury in determining outcome after aSAH. The basis for this was the finding that clazosentan significantly reduced aVSP but did not affect clinical outcome [10]. There are, however, multiple other explanations [9]. Drug side effects/risks can counterbalance its benefits, especially when patients get clazosentan and never develop aVSP and therefore cannot benefit from the drug. The sample size of CONSCIOUS-1 was not designed to affect clinical outcome. The clinical outcome scales might be insensitive to clinically meaningful benefits and there may be practice misalignment. If rescue therapy is effective, it could improve the outcome in the placebo group if it was used more often in these patients, although this did not seem to be a major factor in CONSCIOUS-1.

CONSCIOUS-1 is one of the few studies where almost every one of more than 400 patients had baseline and follow up DSA and where these were measured and assessed by independent physicians. This allows unbiased assessment of risk factors for aVSP and DCI. For example, despite being a well-known prognostic factor for outcomes after SAH, there was no effect of age on aVSP or DIND in CONSCIOUS-1 [84]. It was concluded that it was likely that differences in baseline characteristics of different age groups and other age-related changes in the brain and vasculature led to prior suggestions of an importance of age in aVSP and DIND.

A full examination of predictors of aVSP and DCI in CONSCIOUS-1 has not been conducted. Electrocardiographic findings were assessed in the CONSCIOUS-1 cohort, and QT prolongation was the most common finding [85]. QT prolongation and tachycardia were associated with aVSP.

We assessed DSA in terms of interobserver variability. All DSA were reviewed by a neuroradiologist and a neurosurgeon. Disagreements were adjudicated by a third reviewer who had to agree with one of the first two reviewers. There was agreement on whether there was aVSP or not in 83% of cases (*kappa* = 0.65). For aVSP severity classified as none/mild, moderate or severe, there was 74% agreement with a *kappa* = 0.52. The association between aVSP and DCI was assessed in the CONSCIOUS-1 cohort, as associations between the two have been suggested to be tenuous and a number of other factors were hypothesized to contribute to DCI. There was a strong association between aVSP and cerebral infarction, with an odds ratio of 9.3 for developing infarction if there is severe aVSP compared to not [15]. It should be noted that the diagnosis of delayed cerebral infarction required aVSP and an infarct with no other obvious cause so the association between severe aVSP and infarction may partly reflect the way infarction was defined. On the other hand, there were very few other delayed infarctions in the CONSCIOUS-1 patients that were not associated with severe aVSP. The findings suggest severe aVSP is generally necessary but not always sufficient to cause delayed cerebral infarction. This is somewhat expected, as mentioned above, because of the definition of DCI in CONSCIOUS-1. Relevant to the lack of DCI in some patients with severe aVSP, it is known that patients may be clinically stable with severe aVSP but that with minor changes such as lowering the blood pressure, DCI and infarction can be precipitated [86]. The lack of improved outcome despite a reduction in aVSP, however, suggests that the relationship is not so simple and that microcirculatory flow reductions, microthromboembolism, and spreading cortical depolarizations perhaps playing a role as well. Because the diagnostic method used was CT scan and not MRI, cerebral infarcts and areas of brain injury were almost certainly underestimated.

It was evident in CONSCIOUS-1 that investigator opinion differed from the central review, particularly in assessment of DIND and infarction due to aVSP [10]. Investigators were more likely to diagnose DIND and delayed cerebral infarction when central review concluded there was no aVSP, although the investigators would sometimes diagnose aVSP in these cases [87]. This accounts for the finding that in contrast to investigator opinion, central review showed trends to reduced DIND and delayed cerebral infarction in CONSCIOUS-1 [10]. The lack of effect of clazosentan on rescue therapy is consistent also with the investigators diagnosing and treating what they called DIND in the absence of aVSP.

The prevalence of infarcts and hypodensities on CT after SAH is high; 45% in CONSCIOUS-1 [16]. The most common etiologies were secondary to the aneurysm repair procedure and DCI. Any infarct or hypodensity, regardless of the etiology, must contribute to poor outcome and are therefore targets for prevention. Delayed cerebral infarctions or infarcts secondary to DIND are particularly studied for prevention [88]. It is not easy, however, to determine the etiology of some hypodensities that occur on CT after SAH. The CONSCIOUS-1 data demonstrated that interobserver variability in diagnosis of hypodensities on CT attributed to DIND was considerable (*kappa* = 0.51–0.78; 95% CI, 0.35–0.90) [87]. Patients with hypodensities attributed to DIND were significantly more likely to have severe aVSP, but 19% of these patients had mild or no aVSP. CT hypodensities had a sensitivity and specificity of 41% and 93%, respectively, in identifying patients with severe aVSP, even with expert consensus that these represented aVSP-related delayed cerebral infarcts.

This loose association between aVSP and infarctions was analyzed in the C1 data using path analysis implemented by structural equation modeling to determine direct and indirect path coefficients [88]. Of the 194 patients with moderate to severe aVSP, 43% had neurological worsening of any cause, 20% had cerebral infarction, and 46% poor outcome. Roughly half of patients with moderate/severe aVSP developed neurological worsening and a third of these had infarctions. Only 10% of the remaining half with moderate/severe aVSP who did not show worsening neurological developed infarcts. In patients with none or mild aVSP, 15% had neurological worsening and only 3% had infarcts. Path coefficients showed direct effects on poor outcome from worse WFNS grade, history of hypertension, aVSP, neurological worsening and cerebral infarction. Cerebral infarction contributed to poor outcome by vasospasm-dependent and -independent effects. This suggests that other coexisting factors might be involved in the pathogenesis of DCI.

## 6. Outcomes

Among the explanations for the reduction in aVSP without a commensurate improvement in outcome on the extended GOS in CONSCIOUS-1 is limited sample size. Regulatory approval of drugs in most jurisdictions, at least in the neurologic realm, requires improvement on a patient-centered outcome (mRS, GOS for example) that makes the patient feel, function or survive better [14,43]. These scales are typically collapsed from five or more categories into a dichotomous good or poor outcome, with resulting loss of information and statistical power [89]. Saver described other methods of analysis including global statistic, responder analysis, shift analysis (analysis of distributions, rank analysis, analysis over levels). We compared sliding dichotomy, a type of responder analysis, versus fixed dichotomy of outcome scales in the tirilazad trials, CONSCIOUS-1, and the ISAT cohorts [90]. A sliding dichotomy analysis did not provide improved power and potentially reduce study sample sizes in these patient cohorts. Numerous other analytic and statistical methods exist but they have seldom been applied to SAH RCT and not to CONSCIOUS-1 data [91,92].

A systematic review of outcome measures used in 129 aSAH RCT found the most common functional outcomes among at least 10 scales were the GOS and mRS [93]. A criticism of the mRS and GOS are they do not directly assess cognitive, executive and other neuropsychologic functions that often are affected by aSAH. CONSCIOUS-1 collected the mini-mental state exam so we determined the relationship between this endpoint and the mRS [94]. There was not much variation in the mini-mental state exam and more than half the patients scored 29 or 30 out of 30. There was no significant difference between the mini-mental state scores of patients with a mRS of 0, 1 or 2. This suggests that the mini-mental state test may not be useful for assessing aSAH outcome. Others have suggested the Montreal cognitive assessment may be better [95,96,97]. The question of the best outcome scale for aSAH is being intensively investigated [98,99].

We used several methods that have seldom been used to investigate aSAH. Patient phenotypes that predict poor neurological outcomes following SAH were identified using principal component analysis [100]. Hemodynamic status, WFNS score, neurological injury and initial neutrophil/leukocyte counts were associated with poor outcomes. Similarly, network analysis and graph theoretical analysis found that DIND, anemia and hypoalbuminemia/hypoproteinemia were critical network hubs, and WFNS score and use of rescue therapy had many connections within the network [101]. Quality of life outcomes following aSAH were assessed using a partial least-squares approach [102]. An important disadvantage in almost all previous studies aimed at understanding quality of life following aSAH is multicollinearity among predictor and response variables. In other words, any one dimension of quality of life is often related to others. For example, patients with pain and discomfort may often report anxiety and depression. The predictor variables also are often interrelated. For instance, poor WFNS admission grade is often associated with medical complications, DCI and longer intensive care and hospital stays. Young et al. used the CONSCIOUS-1 data and an advanced, data-driven multivariate approach (partial least squares analysis) to try to disentangle the effects of specific patient phenotypes on quality of life outcomes [102]. Disability in self-care was associated with longer intensive care stay, history of hypertension and worse initial WFNS grade, while disability from pain and discomfort was associated with increased body mass index, longer intensive care stay, worse WFNS grade and higher Hijdra scores.

Weekend admission for aSAH was assessed to determine if there was an association with mortality and long-term neurological outcomes [103]. In the patients with poor admission WFNS grade, weekend admission was independently associated with death. There was no association between weekend admission and three-month neurological outcomes.

Witsch et al. created a prognostic tool to predict long-term functional, cognitive, and quality of life outcomes measures following SAH using an institutional database, entitled the FRESH (Functional Recovery Expected after SAH) score [104]. This score used Hunt and Hess and APACHE-II physiologic scores on admission and age and aneurysmal rebleeding within 48 h. It was externally validated using CONSCIOUS-1, yielding satisfactory prediction ability (area under the curve = 0.77). The CONSCIOUS-1 data have been included in numerous analyses of the SAH international trialists (SAHIT) repository [30].

## 7. Discussion

The CONSCIOUS-1 study made several important contributions. It was the first double-blind, RCT of an endothelin antagonist in aSAH and it was successful [10]. Clazosentan accomplished its pharmacologic action which was to decrease aVSP, which it did in a dose-dependent fashion. It stimulated conduct of two more large, double-blind RCT [11,12]. CONSCIOUS-1 is often cited as responsible for increasing awareness that DCI is a multifactorial complication that only partly depends on aVSP, and for highlighting again the importance of the initial effects of the aSAH (sometimes called early brain injury) on outcome [83,105,106,107]. Recent work suggests that spreading-depolarization-induced cerebral ischemia may also play an important role in DCI after aSAH, with a corresponding drop in cerebral blood flow and tissue partial pressure of oxygen perhaps being regulators of these findings [108]. Although these concepts remain theoretical, they raise the possibility that clazosentan could be used in combination with other therapies to affect aVSP and DCI through multiple pathways.

CONSCIOUS-1 also shows the benefits of well-controlled, double blind experimental type RCT. Similar to ISAT, numerous post-hoc analyses could be conducted on this data prospectively collected from multiple centers with independent verification of endpoints and adverse events. Finally, the CONSCIOUS-1 study stimulated the development of the SAHIT repository [109,110]. Two goals of the SAHIT project were accomplished so far, which were to demonstrate the feasibility of collaborating and sharing data with investigators from around the world and to develop common data elements for RCT [111]. Actelion Pharmaceuticals (now Idorsia Pharmaceuticals Ltd.) must be praised for starting SAHIT by sharing the CONSCIOUS-1 data. Numerous other sets of SAH data have been contributed. A few remain unwilling to share data [112]. Although we possess data from the International Cooperative Study on the Timing of Aneurysm Surgery and some nicardipine RCT, they are stored in SAS version 5 that runs only on Digital Equipment Corporation VAX computers, and efforts to convert this data into newer formats have not been successful [113,114]. Through improving data collaboration efforts, we hope to answer further questions about the pathophysiology, optimal management, and outcomes of patients with aSAH. In particular, characterizing functional outcomes over time, examining medical complications in the era of cooperative neurointensive care teams, determining the efficacy of rescue therapy for aVSP and DCI, and finding new therapies to treat these neurological complications remain important objectives.

To gain a more complete understanding of aSAH management, we recommend reading this paper in conjunction with guidelines statements, that discuss the evidence or lack thereof upon which aSAH management is based [2,56,58,115,116,117].
